# Progression of age-related macular degeneration in eyes with abnormal fundus autofluorescence in a Japanese population: JFAM study report 3

**DOI:** 10.1371/journal.pone.0264703

**Published:** 2022-02-25

**Authors:** Yuji Oshima, Ari Shinojima, Miki Sawa, Ryusaburo Mori, Tetsuju Sekiryu, Aki Kato, Chikako Hara, Masaaki Saito, Yukinori Sugano, Yoshio Hirano, Hitomi Asato, Mayumi Nakamura, Erika Kimura, Mitsuko Yuzawa, Tatsuro Ishibashi, Yuichiro Ogura, Tomohiro Iida, Fumi Gomi, Tsutomu Yasukawa

**Affiliations:** 1 Department of Ophthalmology, Graduate School of Medical Sciences, Kyushu University, Fukuoka, Japan; 2 Department of Medicine, Section of Ophthalmology, Fukuoka Dental College, Fukuoka, Japan; 3 Division of Ophthalmology, Department of Visual Sciences, Nihon University School of Medicine, Tokyo, Japan; 4 Department of Ophthalmology, Keio University School of Medicine, Tokyo, Japan; 5 Department of Ophthalmology, Osaka University Graduate School of Medicine, Suita, Japan; 6 Department of Ophthalmology, Fukushima Medical University School of Medicine, Fukushima, Japan; 7 Department of Ophthalmology and Visual Science, Nagoya City University Graduate School of Medical Sciences, Nagoya, Japan; 8 Department of Ophthalmology, Graduate School of Medicine and Faculty of Medicine, Akita University, Akita, Japan; 9 Santen Pharmaceutical Co., Ltd., Ikoma, Japan; 10 Santen-SERI Open Innovation Centre, Singapore, Singapore; 11 Department of Ophthalmology, Tokyo Women’s Medical University, Tokyo, Japan; 12 Department of Ophthalmology, Hyogo College of Medicine, Nishinomiya, Japan; University of California Los Angeles, UNITED STATES

## Abstract

**Purpose:**

To evaluate the progression of early age-related macular degeneration to neovascular age-related macular degeneration (nAMD), and identify the abnormal fundus autofluorescence (FAF) patterns and markers of choroidal neovascularization (CNV) in fellow eyes of patients with unilateral nAMD.

**Methods:**

Sixty-six patients with unilateral nAMD who developed abnormal FAF in the fellow eyes were enrolled in this multicenter, prospective, observational study, and followed-up for 5 years. FAF images on Heidelberg Retina Angiogram Digital Angiography System (HRA) or HRA2 were classified into eight patterns based on the International Fundus Autofluorescence Classification Group system. The patients in which the fellow eyes progressed to advanced nAMD, including those who did not develop nAMD, were assessed based on the following factors: baseline FAF patterns, age, sex, visual acuity, drusen, retinal pigmentation, baseline retinal sensitivity, family history, smoking, supplement intake, hypertension, body mass index, and hematological parameters.

**Results:**

Of the 66 patients, 20 dropped out of the study. Of the remaining 46 patients, 14 (30.42%, male: 9, female: 5) progressed to nAMD during the 5-year follow-up. The most common (50% eyes) FAF pattern in the fellow eyes was the patchy pattern. According to the univariate analysis, CNV development was significantly associated with age, supplement intake, and low-density lipoprotein levels (p<0.05). Multivariable analysis revealed that patients who showed non-compliance with the supplement intake were more likely to develop nAMD (p<0.05). No significant association was found between the patchy pattern and CNV development (p = 0.86).

**Conclusion:**

The fellow eyes (with abnormal FAF) of patients with unilateral nAMD may progress from early to advanced nAMD. However, no FAF pattern was found that predicted progression in nAMD.

## Introduction

Age-related macular degeneration (AMD) is a progressive retinal degenerative disease, and a leading cause of visual impairment and blindness in older adults. AMD is characterized by the presence of drusen in the macula, followed by choroidal neovascularization (CNV) and macular atrophy [1,2]. Although the etiology of AMD remains largely unknown, environmental factors and genes involved in lipid transport and metabolism, complement cascade, remodeling of the retinal extracellular collagen matrix, and angiogenesis pathways are suggested to be associated with development of AMD [3]. Anti-vascular endothelial growth factor (VEGF) therapy is the current gold standard in the treatment of neovascular AMD (nAMD) associated with CNV. It is known to effectively improve and sustain the visual acuity in most cases of advanced nAMD [4–7]. However, repeated administration of anti-VEGF injections may increase the risk of ocular and systemic complications [8–12].

Previous studies have reported that bilateral dry AMD carries a significant risk of geographic atrophy (GA) and wet AMD development, and therefore, a substantial risk of CNV in the fellow eyes [13–15]. A Japanese population-based study reported that early age-related macular degeneration was a significant risk factor in the progression of nAMD [16].

Fundus autofluorescence (FAF) originates from a complex mixture of bisretinoid fluorophores accumulated in the retinal pigmented epithelium cells as lipofuscin [17]. The abnormal FAF patterns in eyes with early to intermediate AMD are useful in evaluating the progression of AMD [18]. Previously, we identified racial differences in the presentation of late AMD and abnormal FAF patterns that tended to change over time. In another study, we evaluated the association between retinal sensitivity and abnormal FAF, and found that retinal sensitivity near abnormal FAF tended to deteriorate after the third year [19,20].

Since there are no definitive treatments for nAMD, timely management is necessary to prevent the progression of early to late AMD. Therefore, this study aimed to evaluate the progression of early age-related macular degeneration to nAMD, and identify the abnormal FAF patterns and markers of CNV development in fellow eyes of patients with unilateral nAMD. In this study, the analysis was performed using the same subjects as the previously reported JFAM study [19,20].

## Materials and methods

### Study design

This prospective study was conducted by the Japanese Fundus Autofluorescence and Microperimetry in Early Age-Related Maculopathy study group from December 2006 to March 2014, at five university hospitals in Japan. Patients with unilateral nAMD who developed abnormal FAF patterns in the fellow eyes were evaluated. In this study, we used the Age-Related Eye Disease Study (AREDS) system for the classification of AMD [21]. Advanced AMD (AREDS category 4) was characterized by one or more of the following in one eye: GA of the retinal pigmented epithelium involving the foveal center or neovascular maculopathy. In this study, the analysis was performed using the same subjects as the previously reported JFAM study [19,20].

### Ethical statement

This study adhered to the tenets of the Declaration of Helsinki. The Institutional Ethics Committees of the Nagoya City University Graduate School of Medical Sciences, Nihon University School of Medicine, Osaka University Graduate School of Medicine, Fukushima Medical University School of Medicine, and Kyushu University Graduate School of Medical Sciences reviewed and approved the study protocol (University Hospital Medical Information Network approval number: R000043372/UMIN000038050). Written informed consent was obtained from all patients enrolled in the study.

### Study patients and protocol

Sixty-six Japanese patients (age ≥50 years) with unilateral advanced nAMD were enrolled in this 5-year study. The inclusion criteria were the presence of hyperfluorescent or hypofluorescent areas in the fellow eyes on FAF imaging that we have described before [19,20]. Briefly, the inclusion criterion for the study eye was hyperfluorescence or hypofluorescence on FAF images measured by the Heidelberg Retina Angiogram Digital Angiography System (HRA) or HRA2 (Heidelberg Engineering, Heidelberg, Germany). The exclusion criteria were exudative findings in the fellow eyes, such as CNV, hemorrhage, serous retinal pigmented epithelium detachment, serous retinal detachment, hard exudates, diabetic retinopathy, uveitis, high myopia (< -8.0 diopters), retinal vein occlusion, hazy media (interferes with fundus examination), and a history of laser photocoagulation. In this study, 65 fellow eyes had drusen and/or pigmentary abnormalities and 1 fellow eye had extrafoveal GA at the initial visit [19].

All patients underwent an ophthalmologic examination including best-corrected visual acuity (BCVA) analysis, binocular fundoscopy, and color fundus photography at baseline and at the 3-, 6-, 9-, 12-, 24-, 36-, 48-, and 60-month follow-up. FAF imaging and microperimetry (for retinal sensitivity) were performed at baseline and at the 6-, 12-, 24-, 36-, 48-, and 60-month follow-up [19]. CNV development was confirmed by binocular fundoscopy, color fundus photography, optical coherence tomography. To determine the origin of neovascularization, we performed additional OCT, fluorescein and indocyanine green angiography. The patients were interviewed about their smoking habit and supplement intake at the end of the follow-up period or at CNV development. The participants who uses supplement interviewed the product name and ingredients, and also individually asked whether they were taking the supplement daily or intermittently for study period. The fellow eyes that progressed to nAMD were treated at the discretion of the principal investigator.

### Image analysis

The FAF patterns were classified by four authors (MS, TS, YO, and TY) on the basis of the International Fundus Autofluorescence Classification Group system (IFAG) [18–20,22] as follows: minimal change, focal increase, focal plaque-like, patchy, linear, lace-like, reticular, and speckled. The flow chart developed by the Japanese Fundus Autofluorescence and Microperimetry in Early Age-Related Maculopathy study group was used to evaluate the FAF patterns [19]. Briefly, three independent observers evaluated all images and classified the findings into eight patterns: minimal change, focal increase, focal plaque-like, patchy, linear, lace-like, reticular, and speckled. The JFAM study group created a flow chart to facilitate pattern classification that was based on the IFAG definition. We used a flow chart to evaluate the FAF patterns. When an observer’s decision differed from the others, the decision of the two observers who agreed was recorded. When the opinions of the three observers differed, a fourth observer’s decision was considered.

### Statistical analysis

All statistical analyses were performed using JMP Pro software, version 12.0 (SAS, Inc., Cary, NC, USA). The appropriate descriptive statistics (mean, standard deviation, median, range, and percentages) were applied. The analysis of variance, Fisher’s exact test, Chi-square test were used to analyze the correlations between the variables (baseline FAF patterns, age, sex, BCVA, AMD type of the fellow eye, drusen, retinal pigmentation, retinal atrophy, baseline retinal sensitivity, family history, smoking habit, supplement intake, hypertension, body mass index, hematological parameters). Multivariable logistic-regression models were prepared to estimate the risk of CNV development associated with potential predictors including age, LDL cholesterol and supplementation. Inclusion of variables in the models was based on factors that were significant in univariate analysis. We estimated a logistic model which contained all covariates as possible confounders and all interaction terms between perceived patient demand and the covariates as possible effect modifiers. The statistical significance was set at p<0.05.

## Results

### Patient characteristics

**Fig 1:**
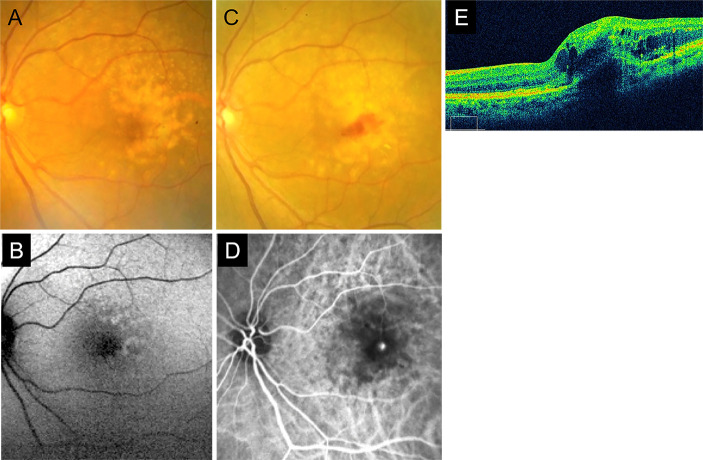
A representative case of retinal angiomatous proliferation. A case of a 70-year-old woman that progressed to type 3 macular neovascularization (MNV). Fundus photograph (A) and fundus autofluorescence (FAF) image (B) at baseline. Confluent drusen (A) and patchy patterns of abnormal FAF (B) were confirmed. At the 6-month follow-up, the fellow eye progressed to type 3 MNV. Intraretinal bleeding, choroidal neovascularization, intraretinal and subretinal fluid, and pigment epithelial detachments were confirmed by fundus photography (C), fluorescein angiography (D), and optical coherence tomography (E).

Sixty-six fellow eyes with abnormal FAF patterns were enrolled in this study. The baseline color fundus photographs of 62 eyes and FAF images of 66 eyes were of sufficient quality to allow accurate assessments [19]. Twenty patients in which fellow eyes did not progress to nAMD dropped out of the study. Of the remaining 46 eyes, nAMD developed in 14 eyes (30.4%, 9 male and 5 female). Type 1 & 2 macular neovascularization (MNV) (7 eyes, 50%) and type 3 MNV (7 eyes, 50%) were the types of nAMD observed in the study. Polypoidal choroidal vasculopathy (PCV) was not reported during the observation period. The average duration (from baseline) for the development of nAMD was 19.9 ± 3.5 months (mean ± standard deviation) (3–42 months, median 15.5 months) (Fig 1, Table 1).

**Table 1:** Baseline profile of patients and characteristics of eyes that progressed to neovascular AMD.

Case	Age (years)	Sex	AMD type of the first affected eyes	FAF pattern	Duration of observation (months)	Type of progressed nAMD (study eye)
1	77	M	PCV	Linear	12	type 1&2 MNV
2	88	M	PCV	Patchy	17	type 3 MNV
3	70	F	type 1&2 MNV	Patchy	6	type 3 MNV
4	72	M	type 1&2 MNV	Patchy	35	type 1&2 MNV
5	75	M	type 1&2 MNV	Focal increase	3	type 1&2 MNV
6	81	M	type 1&2 MNV	Patchy	30	type 3 MNV
7	83	F	type 1&2 MNV	Focal increase	12	type 1&2 MNV
8	69	F	PCV	Patchy	42	type 3 MNV
9	80	F	type 1&2 MNV	Minimal change	14	type 1&2 MNV
10	81	M	type 3 MNV	Patchy	12	type 3 MNV
11	80	M	type 1&2 MNV	Reticular	22	type 3 MNV
12	80	M	type 1&2 MNV	Focal increase	42	type 3 MNV
13	77	F	type 1&2 MNV	Patchy	26	type 1&2 MNV
14	60	M	PCV	Lace-like	6	type 1&2 MNV

AMD, age-related macular degeneration; F, female; FAF, fundus autofluorescence; M, male; nAMD, neovascular age-related macular degeneration; PCV, polypoidal choroidal vasculopathy; MNV, macular neovascularization.

### Fundus findings

FAF images (66 eyes) of sufficient quality were obtained at baseline and classified into eight patterns according to the International Fundus Autofluorescence Classification Group classification system [19,20]. The baseline FAF patterns of eyes that progressed to CNV (n = 14) during the study period were as follows: focal increase, 3 (22%); lace-like, 1 (7%); linear, 1 (7%); minimal change, 1 (7%); patchy, 7 (50%); reticular, 1 (7%). The baseline FAF patterns of eyes without CNV development eyes (n = 32) were as follows: focal increase, 12 (39%); lace-like, 2 (6%); linear, 2 (6%); minimal change, 1 (3%); patchy, 11 (34%); reticular, 1 (3%); speckled, 2 (6%); and focal plaque-like, 1 (3%). No significant differences were seen in the baseline FAF patterns (p = 0.86) in eyes with and without CNV development (Table 2).

**Table 2:** Baseline abnormal FAF patterns in eyes with and without CNV.

Abnormal FAF pattern	Eyes with CNV development (n = 14)	Eyes without CNV development (n = 32)	p value
Focal increase	3 (22%)	12 (39%)	0.8648
Lace-like	1 (7%)	2 (6%)	
Linear	1 (7%)	2 (6%)	
Minimal change	1 (7%)	1 (3%)	
Patchy	7 (50%)	11 (34%)	
Reticular	1 (7%)	1 (3%)	
Speckled	0	2 (6%)	
Focal plaque-like	0	1 (3%)	

CNV, choroidal neovascularization; FAF, fundus autofluorescence.

### Predictive factors for CNV development

The abnormal FAF patterns and predictive factors (Table 3) for CNV development in fellow eyes of patients with unilateral nAMD were identified. The variables assessed were as follows: baseline FAF patterns, age, sex, BCVA at baseline, drusen (soft and hard), pigmentary abnormalities, mean retinal sensitivity at baseline, family history, smoking, supplement intake, hypertension, body mass index, and hematological parameters (Hemoglobin A1c, glucose, uric acid, total cholesterol, triglycerides, high-density lipoprotein cholesterol level, and low-density lipoprotein cholesterol level). According to the univariate analysis, patients with and without CNV development showed significant differences in age (p = 0.0171), supplement intake (p = 0.0362), and low-density lipoprotein cholesterol level (p = 0.0434). The supplements products that were taken continuously were Ocuvite (Bousch & Lomb Japan Co., Ltd., Tokyo, Japan) or Sante Lutax (Santen Pharmaceutical Co., Ltd., Osaka, Japan). The supplements were taken in tablet form 3 times a day after each meal, contained 20 mg of lutein, 300–408 mg of vitamin C, 150–241 mg of vitamin E, 15–30 mg of zinc, and 1.2–1.5 mg of copper as total daily dosages for study period.

**Table 3:** Predictive factors for CNV development.

Factors	Eyes with CNV development (n = 14)	Eyes without CNV development (n = 32)	p value
Age (years)	76.6	71.0	0.0171^†^*
Sex (Male)	9 (64%)	22 (69%)	0.767^‡^
BCVA (logMAR)	1.1 (-0.027)	1.1 (-0.027)	0.998^†^
Diagnosis of the first affected eye			
• Type 1&2 MNV • PCV • 3 MNV	9 (64%) 4 (29%) 1 (7%)	22 (69%) 10 (31%) 0	0.419^‡^
Soft drusen	12 (85%)	30 (93%)	0.298^‡^
Hard drusen	5 (36%)	9 (28%)	0.491^‡^
Pigmentation	7 (50%)	13 (41%)	0.499^‡^
Depigmentation/Atrophy	2 (14%)	9 (28%)	0.463^‡^
Mean retinal sensitivity (dB)	12.9	14.3	0.161^†^
Family history	1 (9%)	3 (11%)	0.854^‡^
Smoking habit			
• Current • None • Former	0 6 (46%) 7 (54%)	4 (16%) 11 (42%) 11 (42%)	0.437^‡^
Supplement intake	5 (38%)	19 (73%)	0.0362*^‡^
Hypertension	3 (60%)	9 (53%)	0.779^‡^
BMI	23.3	23.6	0.717^†^
HbA1c (%)	5.3	5.5	0.289^†^
Glucose (mg/dl)	114	115	0.948^†^
Uric acid (mg/dl)	5.2	5.4	0.579^†^
Total cholesterol (mg/dl)	198.8	215.8	0.151^†^
Triglycerides (mg/dl)	159	161	0.948^†^
HDL cholesterol (mg/dl)	58.7	56.4	0.578^†^
LDL cholesterol (mg/dl)	113	132	0.0434*^†^

BCVA, best-corrected visual acuity; BMI, body mass index; CNV, choroidal neovascularization; FAF, fundus autofluorescence; HbA1c, Hemoglobin A1c; HDL, high-density lipoprotein; logMAR, logarithm of the minimum angle of resolution; LDL, low-density lipoprotein; PCV, polypoidal choroidal vasculopathy; MNV, macular neovascularization.

* p<0.05

^†^analysis of variance

^‡^ chi-square test or Fisher’s exact test.

Multivariable analysis revealed that non-compliance with supplement intake were independent risk factors for nAMD and CNV development (p<0.05) (Table 4).

**Table 4:** Multivariable-adjusted ORs of risk factors for CNV development.

Risk factor	OR	95% CI	p value
Age (per 1 year)	1.096	0.962–1.281	0.1697
LDL cholesterol (mg/dl)	0.981	0.934–1.022	0.3846
Supplement not intake	9.510	1.196–204.97	0.032*

CI, confidence interval; OR, odds ratio

* p<0.05, multiple logistic regression analysis.

## Discussion

This 5-year observational study aimed to evaluate the progression of early age-related macular degeneration to nAMD in fellow eyes of patients with nAMD. In this prospective study, 30.4% of the patients developed nAMD in the fellow eyes. The rate of CNV progression in the fellow eyes of patients with unilateral AMD has been studied by several investigators [23–27]. Cachulo et al. conducted a 2-year prospective study to evaluate the rate of CNV development in patients with unilateral nAMD and early age-related macular degeneration in the fellow eyes. They reported that 32.7% of the patients developed nAMD in the fellow eyes at 2 years [27]. The submacular surgery trials research group reported the 2-year (22%) and 4-year (37%) cumulative incidence rates of CNV development in patients with unilateral subfoveal CNV secondary to nAMD. They concluded that frequent angiography of fellow eyes at risk for CNV development may lead to early detection and timely management of nAMD, and therefore, better visual acuity outcomes [28]. Joachim et al. evaluated the 5-year progression from unilateral to bilateral AMD using data from 3 population-based cohort studies (Blue Mountain Eye Study, Beaver Dam Eye study, and Rotterdam study) and reported a conversion rate of 27%–68% in patients with advanced unilateral AMD [29]. Ueta et al. studied the development of typical AMD and PCV in fellow eyes of Japanese patients with exudative AMD and reported the cumulative incidence rates of 3.4%, 9.3%, and 11.3% in 1, 3, and 5 years, respectively [24]. The differences in the progression rates between our study (30.4%) and the previous reports may be attributed to the study design and the inclusion of abnormal FAF patterns in our study.

In this study, we assessed the accuracy of the FAF patterns in predicting CNV development. The patchy FAF pattern was most commonly (50%) observed in eyes that progressed to CNV. However, no FAF patterns at baseline were significantly associated with the development of CNV during the study period. Consistent with our findings, Cachulo et al. reported no significant association between abnormal baseline FAF patterns and CNV development [27]. Smith et al. investigated the autofluorescence characteristics of fellow eyes in patients with AMD and observed a strong correlation between reticular hypo-autofluorescence and pseudodrusen, and CNV development [30]. Einbock et al. investigated the FAF patterns and disease progression in 125 patients with soft drusen and suggested that the patchy FAF pattern was associated with a higher risk of preliminary CNV development [31]. Batolu et al. et al. investigated FAF patterns in patients with nonexudative AMD and to test if FAF patterns affect the development of CNV. They reported that 22 of 178 (12.3%) eyes with nonexudative AMD developed exudative changes after a mean follow-up of 29.2 months. And the most frequent pattern for CNV development was the patchy pattern in 30.4%, followed by linear in 25%, and reticular pattern in 20.8% of eyes [32].

In this study, the color fundus photographs of 44 fellow eyes were evaluated (photographs of 2 eyes were of insufficient quality) and soft drusen was observed in 37 eyes. Of the 14 eyes that progressed to CNV, 7 eyes (50%) developed typical AMD and 7 eyes (50%) developed type 3 MNV. As compared to a previous study, our study observed a high rate of type 3 MNV development [33]. No cases progressed to PCV in this study. It is reported that some PCVs may fall within the “pachychoroid spectrum diseases” and are associated with choroidal thickening without drusen [34]. This may be one of the reasons that no progression to PCV was observed in this study as drusen was found in most cases.

In the present study, non-compliance with the prescribed supplements was independent risk factor for CNV development. Age was not an independent risk factor in multivariable analysis, but univariable analysis showed a tendency. Yasuda et al. conducted a 9-year study in Japanese individuals and reported old age as a risk factor (odds ratio, 1.10 per year) for development of nAMD [16]. Another study (AREDS) reported the effectiveness of zinc and antioxidant supplements in preventing advanced nAMD (category 4: extensive intermediate-size drusen, at least one large drusen, non-central GA in one or both eyes, or advanced AMD or vision loss due to AMD in one eye) [15]. In our study, the odds ratio for CNV development in patients who did not consume or adhere to supplement (AREDS formula) regimen was 9.510 as compared to patients who complied with the supplement intake. These results indicate that continuous supplement intake is effective in preventing CNV development in patients with unilateral nAMD and abnormal FAF patterns in the fellow eyes.

The current study had some limitations. First, the sample size of the study (number of eyes) was small, and the dropout rate of this study is high. It is supposed that this study may have selection bias, because it cannot be denied that no one in the dropout patient has developed nAMD. Second, the role of genetic factors in the progression of AMD was not considered. Therefore, large scale studies with longer follow-up periods, that take into account the genetic etiology of AMD are needed to understand the association between abnormal FAF patterns and CNV development.

## Conclusions

In this 5-year study, fellow eyes (with abnormal FAF) of patients with unilateral nAMD were evaluated for the development and progression of nAMD. Approximately 30% of the patients progressed to nAMD during the study period. Our results showed that non-compliance with supplement intake was risk factors for nAMD. However, no correlations were found between the baseline FAF patterns and the development and progression of nAMD. Continuous intake of supplements might be a one of factor in reducing the risk of developing CNV in fellow eyes of Japanese patients with unilateral nAMD.
